# Precision of electromyography according to the calibration approach of neuromuscular monitoring: a randomised prospective agreement study

**DOI:** 10.1007/s10877-025-01304-z

**Published:** 2025-05-28

**Authors:** Flora T. Scheffenbichler, Bernhard Ulm, Laura Borgstedt, Anna Scholze, Nadine Kretsch, Nadine Zia, Viola Friedrich, Magdalena Marb, Stefan J. Schaller, Bettina Jungwirth, Manfred Blobner

**Affiliations:** 1 Department of Anaesthesiology and Intensive Care Medicine, Faculty of Medicine, University of UIm, Ulm, Germany; 2 Department of Anaesthesiology and Intensive Care Medicine, TUM School of Medicine and Health, Technical University of Munich, Munich, Germany; 3 Department of Anaesthesiology, Intensive Care Medicine and Pain Medicine, Division of General Anaesthesia and Intensive Care Medicine, Medical University of Vienna, Vienna, Austria

**Keywords:** Calibration, Neuromuscular blockade, Neuromuscular blocking drugs, Neuromuscular monitoring, Electromyography

## Abstract

**Purpose:**

Anaesthesia providers often complain that quantitative neuromuscular monitoring does not accurately assess neuromuscular function, a problem that can be mitigated by appropriate calibration. However, there are only very limited recommendations for the calibration of quantitative neuromuscular monitoring in clinical routine. Therefore, this multicentre prospective agreement study compared the precision of electromyography using three different calibration approaches.

**Methods:**

Sixty patients were assigned to one of three investigational calibration approaches: calibration before anaesthesia induction, calibration during anaesthesia induction, i.e., at loss of consciousness and state entropy < 85, or uncalibrated. All patients received electromyography calibration under deep anaesthesia on the second arm (control as recommended for research). The primary endpoint was the repeatability coefficient, which describes the fluctuation of the following train-of-four (TOF) reading. It therefore provides an estimate of the precision of a measurement method. Secondary endpoints included agreement with control calibration and pain at induction.

**Results:**

The repeatability coefficient at TOF ratios ≥ 0.8 indicated that electromyography monitoring was less precise when TOF readings were uncalibrated (0.124 ± 0.130) or with calibration during induction (0.087 ± 0.104) but was acceptable after calibration before induction (0.075 ± 0.036) compared to those measured after calibration on the contralateral arm (control: 0.072 ± 0.027, 0.061 ± 0.021, and 0.083 ± 0.063, respectively). Recall of pain at anaesthesia induction did not differ between investigational groups.

**Conclusion:**

The findings underline the importance of thoroughly performed calibration for precise TOF readings to reliably exclude residual neuromuscular blockade. Electromyography was most precise when calibration was performed under deep anaesthesia (control). If that approach is not possible in the clinical setting, our data suggest that calibration before anaesthesia induction can be considered if previously discussed with the patient.

**Clinical trial registration:**

Clinical Trials NCT04911088, registered January 6, 2021.

**Supplementary Information:**

The online version contains supplementary material available at 10.1007/s10877-025-01304-z.

## Introduction

Although quantitative neuromuscular monitoring is crucial for decreasing pulmonary complications [1] and is now recommended by international guidelines [2, 3], anaesthesia providers still do not employ it systematically [4]. Frequent complaints are that quantitative neuromuscular monitoring does not allow precise assessment of neuromuscular function and that interpretation of results is difficult [5]. Nevertheless, frequent reasons for failure are incorrect electrode positioning, failure to measure baseline neuromuscular function, inappropriate sensor gain, and inadequate stimulation current. All of this could be improved by adequate calibration of monitoring devices, i.e., the search of the supramaximal stimulus, the adaptation of the gain, and the definition of baseline values.

While there is no recommendation for the calibration of quantitative neuromuscular monitoring in clinical routine, recommendations for good clinical research practice have recently been renewed [6]. They include that calibration should be performed after anaesthesia induction, positioning of the patient, and before administration of a neuromuscular blocking agent. Its success must be confirmed with low variability of the twitch response, for which at least 5 to 6 repeated measurements are recommended [6]. Therefore, the time between loss of consciousness, administration of the neuromuscular blocking drug and securing the airway for atraumatic placement of the tracheal tube is prolonged by this measure. Consequently, this approach is rarely used in clinical routine. In everyday anaesthesia practice, neuromuscular monitoring without calibration is most commonly used, or alternatively, a significantly abbreviated calibration immediately after injection of the hypnotic. The latter, however, coincides with other essential tasks, such as securing the airway and haemodynamic stabilisation, which is why it is out of the anaesthesia provider’s focus. Calibration in the awake patient is another alternative but is considered undesired because it causes pain, and the quality of baseline measurements may be affected by involuntary movements [7].

Therefore, this multicentre prospective agreement study compared the precision of electromyography (EMG) using three different calibration approaches, i.e., calibration before anaesthesia, during induction of anaesthesia, or uncalibrated, with the precision of calibration recommended for research. The experimental calibration approaches were performed on one arm, while the calibration recommended for research was performed on the contralateral arm of the same patient and served as control. Precision was quantified by the repeatability coefficient of the train-of-four (TOF) ratio before application of rocuronium and after spontaneous recovery of TOF ratio ≥ 0.8. The repeatability describes the fluctuation of the following TOF reading. A higher repeatability coefficient therefore indicates lower precision. The secondary evaluation included the precision of all TOF ratios and the agreement between control calibration and the investigational approaches.

## Methods

We performed a multicentre prospective observational agreement study at two university hospitals in Germany, which was approved by the local ethics committees (Ulm University #229/21; Technical University of Munich #2022-232-S-Art.82-NP) and prospectively registered on clinicaltrials.gov (NCT04911088, 6 January 2021). Written informed consent was obtained from all subjects. The Guidelines for Reporting Reliability and Agreement Studies were followed where applicable to the method investigated (Supplementary Table 1) [8]. Data was protected based on the hospital’s data collection and storage policy.

### Patient cohort

Patients ≥ 18 years, with American Society of Anesthesiologists (ASA) physical status classification ≤ III, a body mass index of 17.5–30 kg m^-2^, undergoing elective non-cardiac surgical procedures in supine position and access to both arms, and requiring general anaesthesia and moderate neuromuscular blockade were enrolled. Patients with any neurologic disease, ambulatory surgery, pregnancy, indication for rapid sequence induction, or allergy to neuromuscular blocking drugs or reversal agents were not included.

### Randomisation and blinding

Block randomisation with blocks of 12 in a 1:1:1 ratio was performed to one of three investigational groups with competitive recruitment between the two study centres, meaning that both centres enrolled as many eligible patients as possible until the target sample size was reached. The randomisation platform used is a secure website that is hosted by the research group and is not accessible to the public. Based on the nature of the investigation, the study personnel responsible for the calibration approach could not be blinded. Conversely, study personnel distributing postoperative questionnaires and conducting statistical analyses, and patients were blinded to the investigational calibration group.

### Anaesthesia

Anaesthesia induction followed the department’s standard of care. Vital sign monitoring (Carescape monitor B650, GE Healthcare, Helsinki, Finland) included monitoring of depth of anaesthesia. After preoxygenation, patients received fentanyl, followed by propofol, which was subsequently used to maintain anaesthesia. The airway was secured with a supraglottic airway device or a tracheal tube.

### Investigational protocol

Quantitative neuromuscular monitoring was performed on both arms using EMG, the recommended “alternative gold standard” to mechanomyography [9]. The device used (NMT electro sensor, GE Healthcare, Helsinki, Finland) has been scientifically validated against mechanomyography [10]. After degreasing and drying the skin, the five Ag/AgCl electrodes (NMT Electrode, Solid gel, GE Healthcare, Helsinki, Finland) were placed on each arm: two stimulation electrodes on the ulnar nerve, one ground electrode on the wrist, the recording electrode over the hypothenar muscle [11] and the neutral electrode on the lateral metacarpophalangeal joint of the fifth finger. Before the start of anaesthesia, the fingers two to five were fixated with adhesive tape to ensure optimal measurement conditions. The arms were positioned at a 40–80° angle; forearms were not moved intraoperatively and were covered with a warming blanket system.

The TOF mode was set to a time interval of 20 s with a pulse width of 200 µs. Calibration consisted of the automated supramaximal stimulation current search and the subsequent reference measurement. In patients assigned to the group “uncalibrated,” a standard current of 60 mA was applied.

**Fig. 1:**
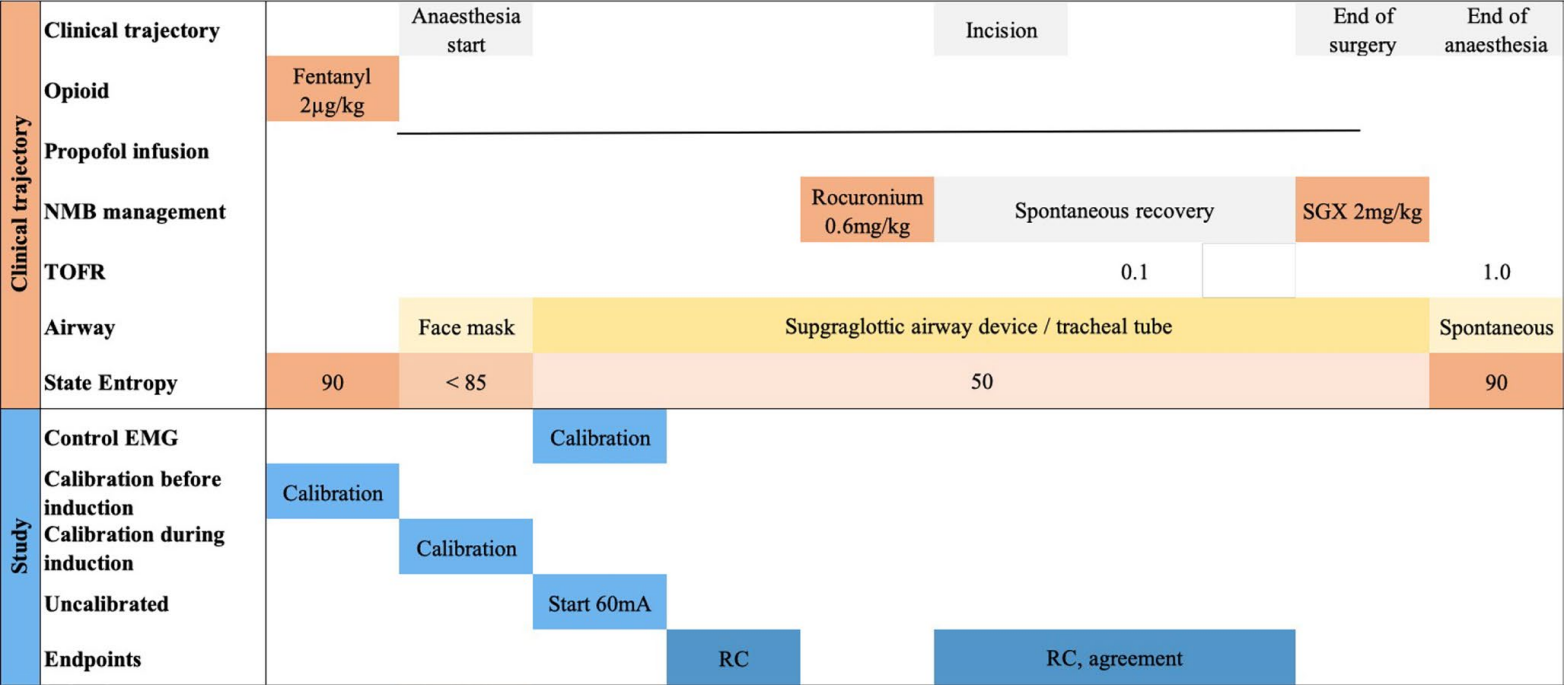
Scheme of clinical trajectory and study protocol of different calibration approaches. EMG, electromyography; NMB, neuromuscular blockade; RC, repeatability coefficient; SGX, sugammadex; TOFR, train-of-four ratio

The investigational calibration “before induction” was performed three minutes after patients received fentanyl 2 µg kg^-1^. The investigational calibration “during induction” was performed after a propofol infusion with 3–5 mg kg^-1^ h^-1^ was started, and patients lost consciousness, i.e., the state entropy dropped below 85 (EasyFit Sensor, GE Healthcare, Helsinki, Finland), and patients did not respond to speech. In patients allocated to the group “uncalibrated”, the EMG was initiated after induction of anaesthesia at a state entropy between 40 and 50. An overview of the clinical trajectory and study protocol is presented in Fig. 1.

Calibration of the EMG as recommended for research (control calibration) [6] was performed on the contralateral arm, i.e., after anaesthesia induction with stable cardiopulmonary conditions (blood pressure, heart rate, or oxygen saturation without treatment requirement) and a state entropy below 50. The number of attempts to achieve successful (automated) calibration, i.e., a stable twitch response to a supramaximal stimulus, was documented. These attempts included repositioning of the electrodes.

After baseline EMG measurements were obtained on both arms, rocuronium 0.6 mg kg^-1^ was administered. At the end of surgery, when spontaneous recovery reached a stable plateau at a TOF ratio of > 0.9 on the EMG which was calibrated as recommended for research, sugammadex 2 mg kg^-1^ was administered (Fig. 1) to guarantee complete neuromuscular recovery independent of the TOF ratio [11] and to exclude recurrence of neuromuscular block [12]. EMG readings were continued until 10 min after administration of the reversal agent.

### Endpoints

The primary endpoint was precision at baseline, i.e., before administration of rocuronium and precision during neuromuscular recovery (TOF ratios ≥ 0.8). Precision was expressed by the repeatability coefficient and compared within patients based on the randomised calibration group. A low repeatability coefficient indicates high precision. Secondary endpoints included agreement between TOF ratios after investigational and control calibration and the time difference to reach a TOF ratio > 0.9 and > 0.95 between calibration techniques. Postoperative secondary endpoints included recall of pain at anaesthesia induction on a visual analogue scale (0-100).

### Sample size

The sample size was calculated based on a two-sided dependent t-test with an effect size of 0.8, a power of 90% and an alpha of 5%. This resulted in a sample size of 19 patients per group. In order to compensate for possible technical difficulties, one patient was added per group resulting in a sample size of 60 patients. Prior to enrolment, 6 roll-in patients were included to optimise data quality and protocol adherence.

### Statistical analysis

Precision was analysed using one-way analysis of variance on sets of consecutive TOF ratios measured by the same calibration approach. The repeatability coefficient is a measure of precision that indicates the range in which the absolute difference between two repeated test results is likely to fall with 95% probability and has been repeatedly used for describing neuromuscular monitoring devices [13–16]. The 95% repeatability coefficient was calculated as $\:1.96\sqrt{2{\widehat{\sigma\:}}_{W}}$, where $\:{\widehat{\sigma\:}}_{W}$ is defined as the square root of the residual mean square.

Individual repeatability coefficients allowed a comparison of the control calibration with the test calibration using the Wilcoxon signed-rank test for paired samples.

Agreement was analysed using Bland-Altman analysis [17]. The limits of agreement were calculated as the 1.96 $\:{\widehat{\sigma\:}}_{D}$, where $\:{\widehat{\sigma\:}}_{D}$is defined as the standard deviation of the difference between the investigational and the control calibration. A Bland-Altman diagram was created for each calibration group.

To be certain that a TOF ratio has exceeded 0.9, repetitions of the measurement are recommended. Assuming the repeated TOF readings are normally distributed, their mean value ($\:\overline{TOF\text{}ratio}$) must meet the following condition to achieve 95% confidence:

$\:\overline{TOF\text{}ratio}\text{>}0.9\text{+}1.96\frac{{\sigma\:}_{i}}{\sqrt{{n}_{i}}}$where n_i_ is defined as the number of TOF readings after TOF ratio > 0.9 of an individual patient and $\:{\sigma\:}_{i}$ as standard deviation of the repetitive TOF readings. As an exploratory outcome, the number of TOF readings until a 95% certainty of a TOF ratio > 0.9 is reached, was calculated using $\:{\sigma\:}_{i}$ of the TOF ratio ≥ 0.8 as the starting point. According to repetitive TOF stimulation every 20 s, the number of necessary TOF readings can be translated in times to be waited before 95% certainty of TOF ratio > 0.9 is given. Log-rank tests, corrected according to the Bonferroni method, were used to compare the Kaplan-Meier curves.

Since the 95% repeatability coefficient (RC_95_) of a cohort of patients is calculated as $\:1.96\sqrt{2\:{\widehat{\sigma\:}}_{W}}$, it can also be used to describe the condition for certainty of neuromuscular recovery in the respective cohort of patients ($\:{\sigma\:}_{i}$ is set equal to $\:{\stackrel{\prime }{\sigma\:}}_{w}$) with the RC_95_:

$\:\overline{TOF\text{}ratio}\text{>}0.9\text{+}\frac{{RC}_{95}}{\sqrt{2{n}_{i}}}$. This relation is used to illustrate the influence of the investigational calibration via precision, i.e., the RC_95_ value, on the number of repetitions.

For all EMG-related outcomes, neuromuscular monitoring data at baseline, as well as those from different pre-defined ranges of neuromuscular recovery, were analysed, as published previously by Liang and colleagues(13). A TOF ratio > 0.9 was considered for exploratory analyses. A p-value of 0.05 was considered significant. All analyses were performed using the statistical software R (version 4.3.3, R Foundation for Statistical Computing, Vienna, Austria).

## Results

**Fig. 2:**
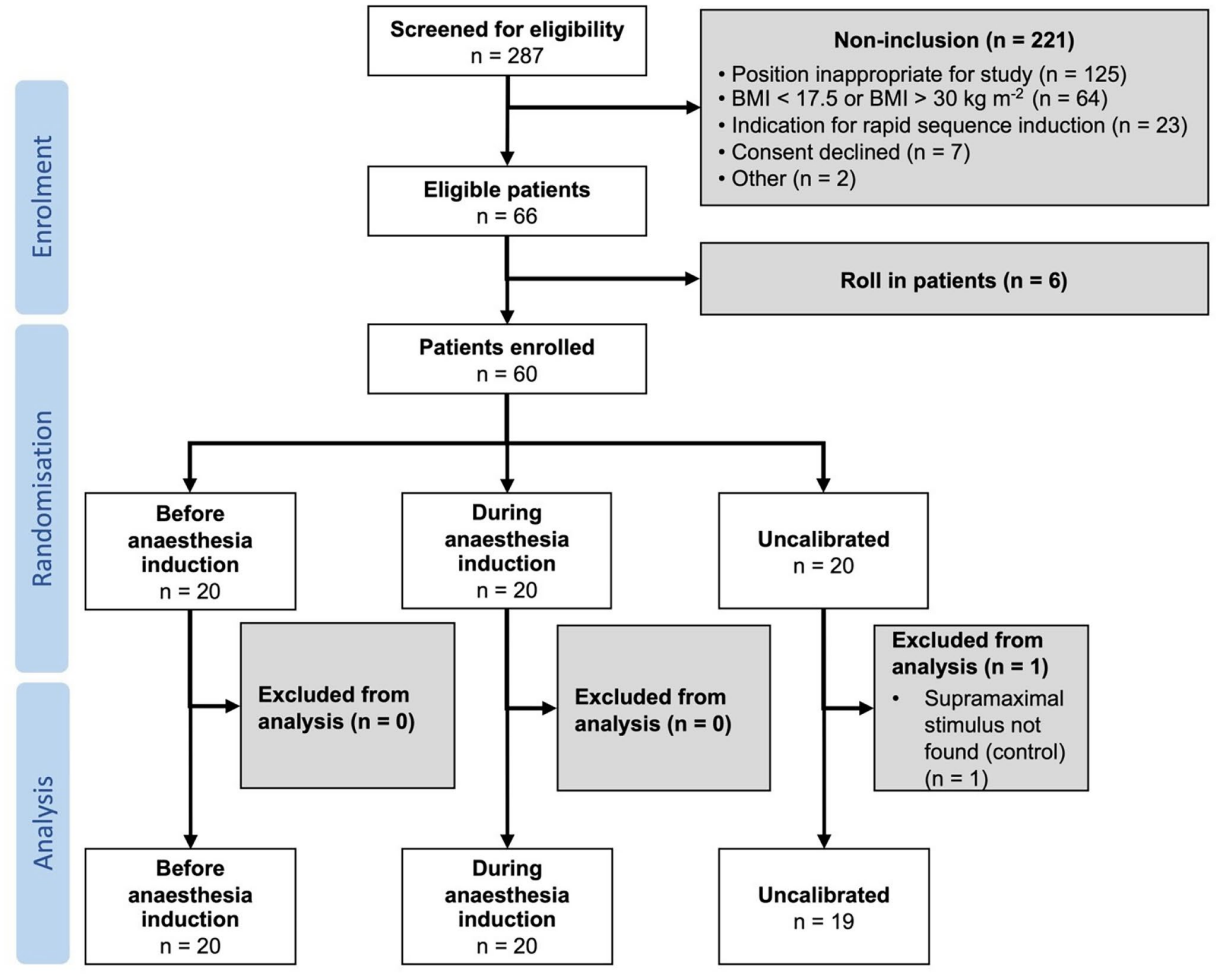
Patients’ recruitment and allocation. BMI, Body Mass Index

Sixty adult patients were enrolled between January 2022 and May 2023 (Fig. 2). The study cohort consisted of 25 female and 34 male patients who were on average 44 (29–63) years old, weighed on average 74 (63–82) kg, and had ASA physical status I (*n* = 29), II (*n* = 23), and III (*n* = 7). Patient characteristics were similar in the calibration groups (see Supplementary Table 2 for details). All patients received rocuronium 0.6 mg kg^-1^ and sugammadex 2 mg kg^-1^.

### Calibration success

Calibration before anaesthesia induction was successful at the first attempt in 19 of 20 patients and after ≥ 2 attempts in 1 of 20 patients. One attempt was necessary to calibrate during anaesthesia induction in 19 of 20 patients, and 1 of 20 needed ≥ 2 attempts. A suboptimal positioning of the electrodes was recognised and corrected in 1 of 20 patients with uncalibrated EMG. Control calibration was successful at the first attempt in 51 patients, at the second attempt in 3 patients, at ≥ 2 attempts in 5 patients, and not possible in 1 of 60 patients. Since the supramaximal stimulus was not found, the patient was excluded. The stimulation current was, on average, 58 ± 12 mA in the calibration group before induction, 55 ± 11 mA in the calibration group during induction, and 57 ± 12 mA in the control group.

### Precision

TOF readings were less precise when EMG monitoring was conducted without calibration or with calibration during anaesthesia induction, but not after calibration before anaesthesia induction. This is demonstrated by higher repeatability coefficients for uncalibrated EMG and for calibration during anaesthesia induction with highest (most unprecise) values at advanced recovery (TOF ratio range ≥ 0.8, Table 1).

**Table 1:** Repeatability coefficient of all pairs of consecutive measurements in patient groups for the calibrations studied and for the calibration as recommended for research (control). The values are provided for baseline (before administration of rocuronium) and during certain recovery periods.

TOF ratio range	Calibration	Investigational calibration group					
		Before anaesthesia induction		During anaesthesia induction		Uncalibrated	
		*n*	RC	*n*	RC	*n*	RC
**Primary Endpoint**							
Baseline	investigation	127	0.055 ± 0.046	124	0.071 ± 0.183	98	0.073 ± 0.080
	control*	104	0.041 ± 0.064	120	0.063 ± 0.189	82	0.024 ± 0.039
≥ 0.80	investigation	1512	0.075 ± 0.036	1534	0.087 ± 0.104	1443	0.124 ± 0.130
	control*	1496	0.083 ± 0.063	1729	0.061 ± 0.021	1439	0.072 ± 0.027
**Secondary endpoints**							
0.60–0.79	investigation	441	0.032 ± 0.026	295	0.051 ± 0.080	354	0.047 ± 0.065
	control*	448	0.026 ± 0.017	379	0.018 ± 0.008	356	0.021 ± 0.008
0.40–0.59	investigation	315	0.025 ± 0.014	240	0.031 ± 0.025	284	0.057 ± 0.093
	control*	330	0.020 ± 0.021	296	0.027 ± 0.032	294	0.029 ± 0.046
0.20–0.39	investigation	337	0.031 ± 0.023	250	0.026 ± 0.020	377	0.053 ± 0.063
	control*	340	0.018 ± 0.010	310	0.021 ± 0.018	403	0.032 ± 0.022
Recovery (≥ 0.20)	investigation	2605	0.071 ± 0.025	2319	0.106 ± 0.107	2458	0.134 ± 0.125
	control*	2614	0.068 ± 0.040	2714	0.084 ± 0.065	2492	0.101 ± 0.072

Data are means ± standard deviation. * Calibration recommended for research was performed under deep anaesthesia on the contralateral arm. Number of consecutive measurements: n; repeatability coefficient: RC; Train-of-four: TOF

The individual means of the repeatability coefficients after control calibration were significantly lower compared to those without calibration (median [interquartile range]: 0.028 [0.020–0.035] vs. 0.035 [0.028–0.080]; median difference 0.014 [95% confidence interval [CI]: 0.006 to 0.050]; *p* < 0.001) as well as compared to calibration during anaesthesia induction (0.028 [0.021–0.040] vs. 0.033 [0.024–0.075]; median difference 0.008 [0.000 to 0.037]; *p* = 0.044), but not compared to calibration before induction of anaesthesia (0.025 [0.018–0.034] vs. 0.037 [0.024–0.045]; median difference 0.008 [-0.003 to 0.020]; *p* = 0.114).

The gauge standard deviation was analysed for baseline TOF readings, confirming the better precision of TOF readings following calibration as recommended for research (Supplemental Table 3).

### Agreement

**Fig. 3:**
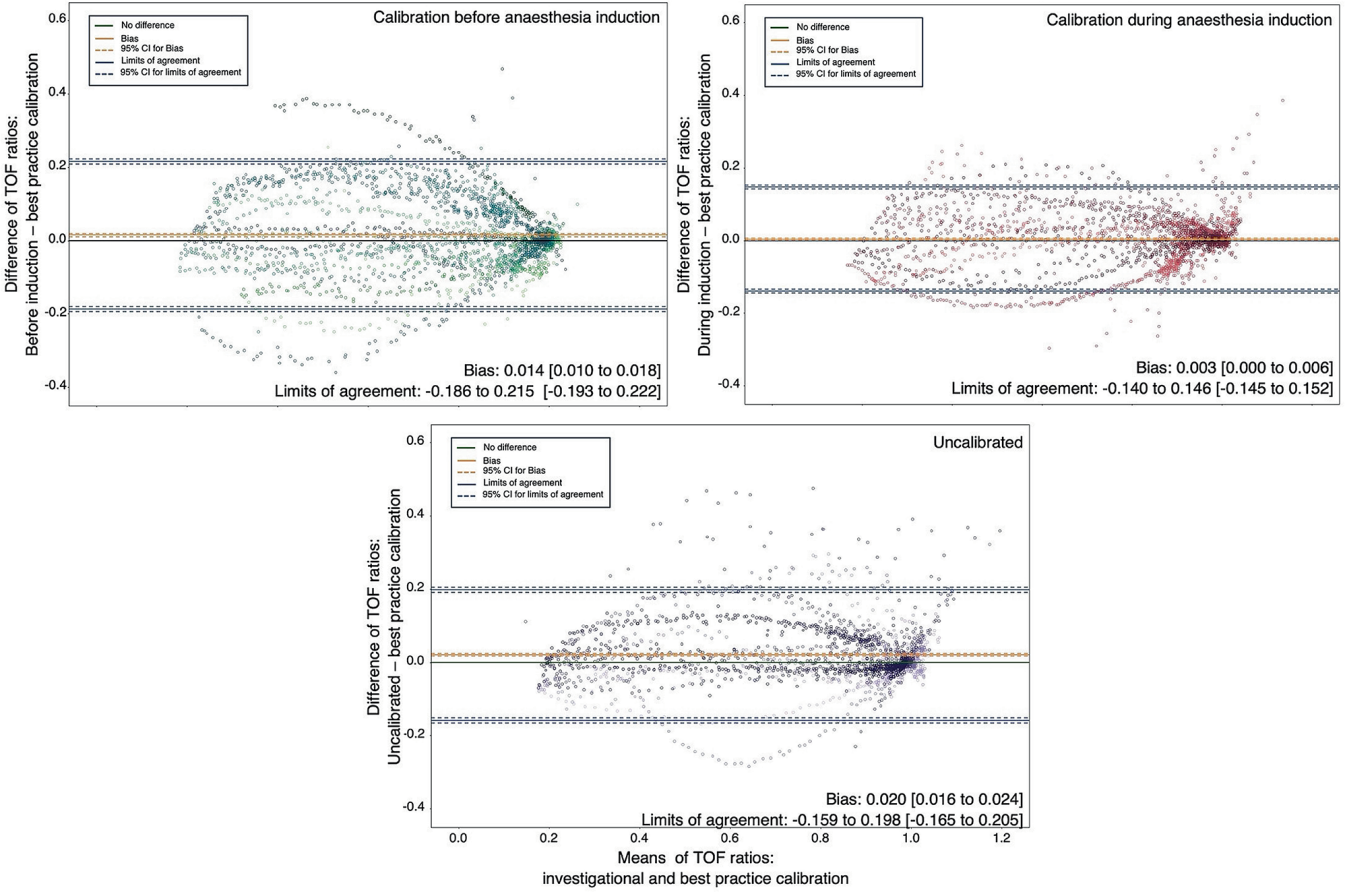
Bland Altman plots of train of four (TOF) ratios during neuromuscular recovery measured after investigational vs. best-practice calibration. Detailed information including bias and limits of agreement are provided in the Supplementary Table 4. Bias [95% confidence interval]. Limits of agreement [outer 95% confidence borders]

At TOF ratios ≥ 0.8, there was no bias between EMG-measured TOF readings after calibration before, or during anaesthesia induction and the control calibration (bias [95 CI]: -0.002 [-0.005 to 0.002 and 0.000 [-0.003 to 0.003], respectively). Uncalibrated measured TOF ratios minimally overestimated the level of recovery (0.008 [0.004 to 0.012]). During the complete neuromuscular recovery, TOF readings measured after all investigational calibration approaches minimally overestimated the level of recovery (Fig. 3 and Supplemental Table 4). Times to a TOF ratio > 0.9 and and a TOF ratio > 0.95 did not differ significantly between investigational and control calibrated monitoring (Supplemental Table 5). Further results are displayed in the supplemental results.

### Patient-centred evaluation

Patients whose EMG was calibrated before anaesthesia induction significantly better recalled involuntary movements (*p* < 0.001) compared to the other calibration groups. There was no difference in perception of pain between groups. Similarly, acceptance of discomfort for safer anaesthesia did not differ (Table 2). One patient left the hospital before completing the postoperative questionnaire.

**Table 2:** Patient experience with the calibration procedure was evaluated using a visual analogue scale ranging from 0 to 100 mm. Data are displayed according to the investigational calibration group. Medians and interquartile ranges are shown.

Questionnaire item	Before anaesthesia induction (*n* = 19)	During anaesthesia induction (*n* = 20)	Uncalibrated (*n* = 19)	*p*-value
Severe pain during anaesthesia induction	10 (7–21)	9 (7–11)	7 (5–10)	0.296
Explicit memory of involuntary movement	92 (18–95)	26 (7–93)	7 (5–10)	< 0.001
Acceptance of discomfort for safety	91 (70–95)	86 (37–93)	72 (11–89)	0.173

### Exploratory considerations

**Fig. 4:**
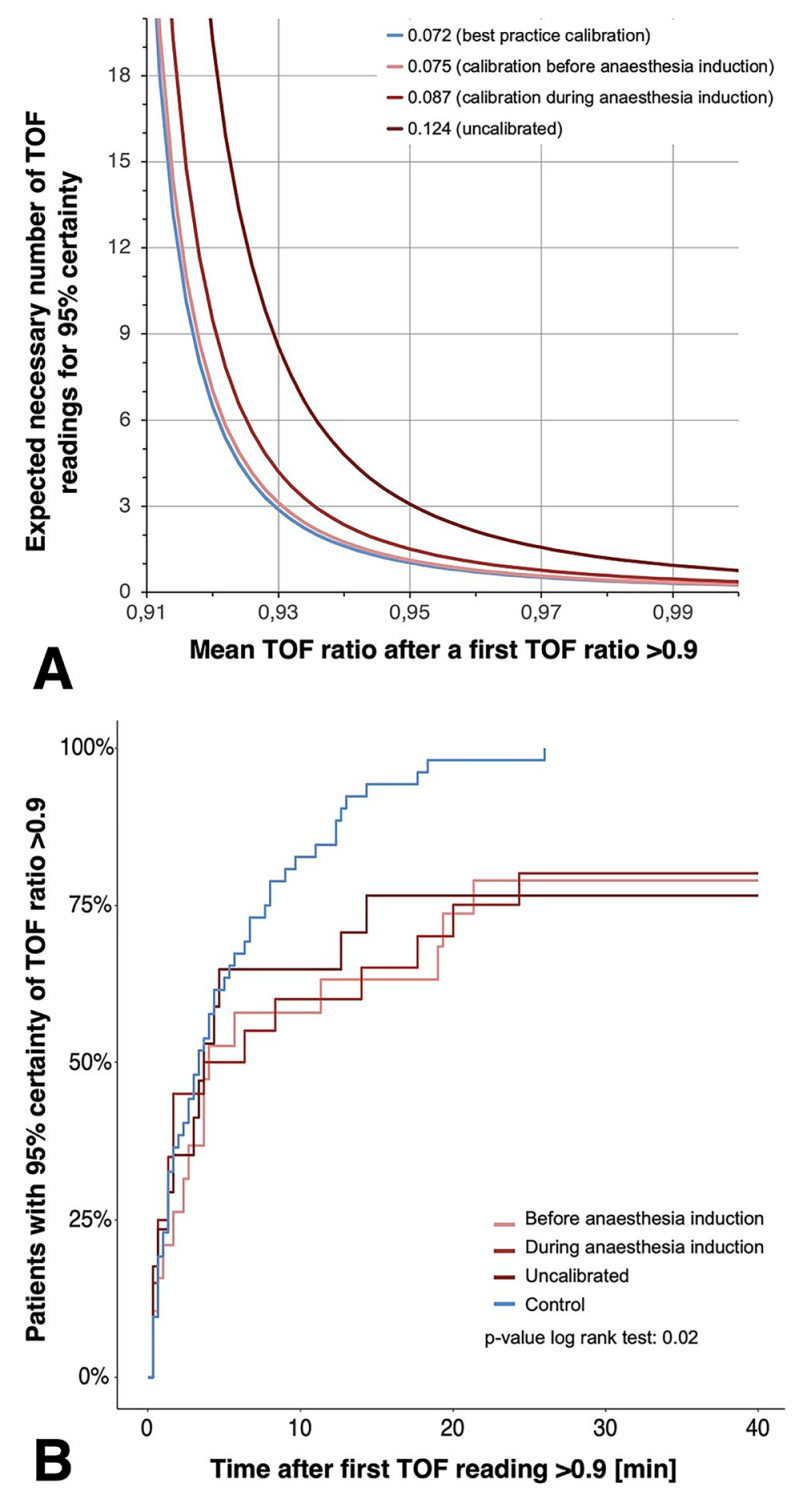
Influence of low precision (high repeatability coefficients [RC]) due to poor calibration on the need for repetitive train-of-four (TOF) readings when aiming for a 95% certainty that the TOF ratio exceeds. **A** shows that high repeatability coefficients (i.e. low precision) require more TOF readings and high mean TOF values, using the repeatability coefficients of the four calibration approaches exemplarily. In **B**, Kaplan-Meier curves show the cumulative percentage of patients with 95% certainty of TOF ratios > 0.9. Low precision due to missing or poor calibration prolonged the necessary monitoring time (Log rank: *p* = 0.02)

In an exploratory analysis, we evaluated the number of TOF readings (translated into the required time) necessary before a TOF ratio > 0.9 can be considered 95% certain based on the individual precision (Fig. 4). According to repetitive TOF stimulation every 20 s, 10 min after reaching a TOF ratio > 0.9, 98.3% of patients achieved a reliable TOF ratio > 0.9 when calibrated as recommended for research, 85% when calibrated before anaesthesia induction, 70% when calibrated during anaesthesia induction, and 79% when EMG was performed uncalibrated. Log rank test revealed a significantly different number of necessary repetitions, i.e. 95% certainty of TOF ratio > 0.9, between the different calibration approaches (p = 0.02).

## Discussion

This multicentre, randomised agreement study shows that precise neuromuscular monitoring requires careful calibration. During neuromuscular recovery, uncalibrated EMG resulted in imprecise TOF readings, indicated by a repeatability coefficient of 0.124 ± 0.130, almost double that of EMG calibrated under conditions recommended for research (0.072 ± 0.027). Of note, the precision with calibration during induction of anaesthesia was markedly better (0.087 ± 0.104) than uncalibrated EMG. Calibration before induction of anaesthesia, however, had the most precise repeatability coefficient of all investigational groups (0.075 ± 0.036). Notably, a repeatability coefficient of 0.12 means that after a TOF reading of 0.90, the subsequent reading under unchanged conditions will fall within the range of 0.78 to 1.02 with 95% certainty. This is highly clinically relevant for correctly interpreting the threshold TOF ratio of 0.9 to reliably rule out residual neuromuscular blockade at the end of anesthesia.

The uncalibrated approach led to the lowest precision, underlining the high importance of calibration. However, due to ambiguous results, even recently published guidelines lack specific recommendations on calibration of quantitative neuromuscular monitoring [2, 3]. Consequently, calibration is rarely performed in clinical practice [18, 19]. It must also be assumed that the high repeatability coefficients found in this study are still rather low because, unlike in everyday clinical practice, EMG monitoring was started before the neuromuscular blocking drug was administered. Suboptimal positioning of the electrodes in the uncalibrated EMG was recognised and corrected in one out of 20 patients, which is likely to be unnoticed when the neuromuscular monitoring is started in paralysed patients. In fact, the retrospectively evaluated median repeatability coefficient in 128,168 patients in one of the two participating departments’ database was 0.403.

The clinically most common calibration approach, i.e., during induction, provided critically imprecise TOF readings. These findings may be explained by uncontrolled movement during calibration despite information about possible involuntary movements of the arm. The patients’ excitation may have led to unstable conditions and, subsequently, less precise TOF readings. In addition, necessary repositioning of the electrodes and recalibration may be impaired due to the time pressure inherent in this calibration method.

The precision of calibration before anaesthesia induction with propofol did not significantly differ from calibration as recommended for research. This calibration approach is usually rejected because it is painful and distorted by movement. When planning the study, we did not expect the high precision found in the study using this calibration method. Had one of the other clinically common methods provided a similar precision, the concerns about calibration in conscious patients would have been enough to reject it.

Unmedicated volunteers reported pain on a verbal numeric rating scale with 5 of 10 points at a stimulation current of 50 mA [7]. In contrast, in the presented study, the calibration before anaesthesia induction but three minutes after intravenous fentanyl (2 µg kg^-1^) and detailed information about the need and the expected benefit of valid neuromuscular monitoring effectively prevented pain despite full consciousness. The questionnaire referred to pre-anaesthesia induction pain in general, which was comparable between the calibration study methods. However, the perception of twitching was increased in the pre-anaesthesia induction group, indicating sufficient recall of the procedure and adequate analgesia. Twitching could be considered as discomfort but would be accepted by most patients in the context of safer anaesthesia. If there are medical reasons against extending the time between loss of consciousness and tracheal intubation, e.g. for rapid sequence intubation, calibration in well-informed, consented patients under sufficient analgesia could be considered based on our data.

In an agreement study published by Liang and colleagues, acceleromyography was compared to the same EMG used in the present study. They revealed that EMG provided more precise TOF readings and quantified the repeatability coefficient to be 0.051, which is comparable to our results [13]. Nevertheless, EMG is rarely used in clinical practice because the positioning of the five electrodes is more complicated compared to only two acceleromyography electrodes. Interestingly, in a study comparing electromyography using disposable electrode patches with acceleromyography, no statistically significant difference was found for the time to connect the devices [20]. The conceptual superiority of EMG over AMG-based neuromuscular monitoring in terms of TOF measurement precision was also confirmed for three EMG devices with disposable electrode patches [21, 22]. So far, no information is available if such patches may also increase precision when calibration is performed during induction of anaesthesia or when not calibrated.

Our simulation of how to obtain certainty that the TOF ratio has exceeded the threshold of 0.9 illustrates that the presumably low precision of monitoring in everyday clinical practice can be compensated. A high mean TOF ratio and a high number of repeated measurements after the threshold is exceeded for the first time can partially compensate for a lack of precision (Fig. 4). In clinical routine, due to time pressure not only during induction but also during emergence from anaesthesia, neuromuscular monitoring is usually terminated, and the patient’s trachea extubated as soon as a TOF ratio of > 0.9 is reached. This may explain why the pulmonary outcome of the patients was not better after TOF ratio > 0.9 but after TOF ratio > 0.95 in the observational POPUALR study [23].

This is to our knowledge, the biggest study investigating the precision of EMG calibration methods. We used an intraindividual comparison between the investigational and the control calibration on each hand to keep the patient-related variability at a minimum [24]. Nevertheless, the study has some limitations. Due to the nature of the investigational calibrations, the study personnel was not blinded. In this study the hypothenar eminence was chosen as the recording site which is equally recommended as the thenar eminence [6]. Furthermore, experimental data suggest that the hypothenar eminence provides the most stable EMG readings [25]. Therefore, it can be expected that using the thenar eminence, higher repeatability coefficients could have been measured. Since we have used the same recording site for the control and the investigational approach, we do not expect this to have any influence on our results. Although EMG is increasingly used as disposable electrodes and recognised as the most appropriate technique for quantitative neuromuscular monitoring [26], some consideration must be given to its artefact filtering. Most EMG devices use voltage-related thresholds to identify artefacts. Therefore, when stimulation currents generate voltages above the threshold, they are not recognised as artefacts. As these artefacts do not fade at deep neuromuscular blockades, erroneously high TOF ratios are displayed. Future EMG developments should use more advanced filtering technology, such as identifying the characteristic shape of the twitch response [27]. Due to the postoperative nature of the questionnaire, pain or discomfort from twitching cannot be separated from, e.g., propofol infusion since both might have occurred at the same time depending on the investigational group.

The findings underline the importance of thoroughly performed calibration for precise EMG TOF readings in clinical practice to reliably exclude residual neuromuscular blockade.

## Electronic supplementary material

Below is the link to the electronic supplementary material.

Supplementary Material 1

## Data Availability

Data is provided within the manuscript or supplementary information files.

## References

[1] Carvalho H, Verdonck M, Cools W, Geerts L, Forget P, Poelaert J. Forty years of neuromuscular monitoring and postoperative residual curarisation: a meta-analysis and evaluation of confidence in network meta-analysis. Br J Anaesth. 2020;125(4):466–82. 10.1016/j.bja.2020.05.063. 32680607 10.1016/j.bja.2020.05.063

[2] Fuchs-Buder T, Romero CS, Lewald H, Lamperti M, Afshari A, Hristovska AM, et al. Peri-operative management of neuromuscular Blockade: A guideline from the European society of anaesthesiology and intensive care. Eur J Anaesthesiol. 2023;40(2):82–94. 10.1097/EJA.0000000000001769. 36377554 10.1097/EJA.0000000000001769

[3] Thilen SR, Weigel WA, Todd MM, Dutton RP, Lien CA, Grant SA, et al. 2023 American society of anesthesiologists practice guidelines for monitoring and antagonism of neuromuscular Blockade: A report by the American society of anesthesiologists task force on neuromuscular Blockade. Anesthesiology. 2023;138(1):13–41. 10.1097/ALN.0000000000004379. 36520073 10.1097/ALN.0000000000004379

[4] Blobner M, Hollmann MW, Luedi MM, Johnson KB. Pro-Con debate: do we need quantitative neuromuscular monitoring in the era of Sugammadex? Anesth Analg. 2022;135(1):39–48. 10.1213/ANE.0000000000005925. 35709443 10.1213/ANE.0000000000005925

[5] Thomsen JLD, Marty AP, Wakatsuki S, Macario A, Tanaka P, Gatke MR, et al. Barriers and aids to routine neuromuscular monitoring and consistent reversal practice-A qualitative study. Acta Anaesthesiol Scand. 2020;64(8):1089–99. 10.1111/aas.13606. 32297659 10.1111/aas.13606 PMC7497053

[6] Fuchs-Buder T, Brull SJ, Fagerlund MJ, Renew JR, Cammu G, Murphy GS, et al. Good clinical research practice (GCRP) in pharmacodynamic studies of neuromuscular blocking agents III: the 2023 Geneva revision. Acta Anaesthesiol Scand. 2023;67(8):994–1017. 10.1111/aas.14279. 37345870 10.1111/aas.14279

[7] Nemes R, Nagy G, Murphy GS, Logvinov II, Fulesdi B, Renew JR. Awake volunteer pain scores during neuromuscular monitoring. Anesth Analg. 2020;130(4):941–8. 10.1213/ANE.0000000000004326. 31348055 10.1213/ANE.0000000000004326

[8] Kottner J, Audige L, Brorson S, Donner A, Gajewski BJ, Hrobjartsson A, et al. Guidelines for reporting reliability and agreement studies (GRRAS) were proposed. J Clin Epidemiol. 2011;64(1):96–106. 10.1016/j.jclinepi.2010.03.002. 21130355 10.1016/j.jclinepi.2010.03.002

[9] Naguib M, Brull SJ, Kopman AF, Hunter JM, Fulesdi B, Arkes HR, et al. Consensus statement on perioperative use of neuromuscular monitoring. Anesth Analg. 2018;127(1):71–80. 10.1213/ANE.0000000000002670. 29200077 10.1213/ANE.0000000000002670

[10] Carter JA, Arnold R, Yate PM, Flynn PJ. Assessment of the Datex relaxograph during anaesthesia and atracurium-induced neuromuscular Blockade. Br J Anaesth. 1986;58(12):1447–52. 10.1093/bja/58.12.1447. 3024688 10.1093/bja/58.12.1447

[11] Baumüller E, Schaller SJ, Chiquito Lama Y, Frick CG, Bauhofer T, Eikermann M, et al. Postoperative impairment of motor function at train-of-four ratio >/=0.9 cannot be improved by Sugammadex (1 mg kg-1). Br J Anaesth. 2015;114(5):785–93. 10.1093/bja/aeu453. 25586724 10.1093/bja/aeu453

[12] Murphy GS, Szokol JW, Avram MJ, Greenberg SB, Shear TD, Deshur MA, et al. Neostigmine administration after spontaneous recovery to a Train-of-Four ratio of 0.9 to 1.0: A randomized controlled trial of the effect on neuromuscular and clinical recovery. Anesthesiology. 2018;128(1):27–37. 10.1097/ALN.0000000000001893. 28953501 10.1097/ALN.0000000000001893

[13] Liang SS, Stewart PA, Phillips S. An ipsilateral comparison of acceleromyography and electromyography during recovery from nondepolarizing neuromuscular block under general anesthesia in humans. Anesth Analg. 2013;117(2):373–9. 10.1213/ANE.0b013e3182937fc4. 23821356 10.1213/ANE.0b013e3182937fc4

[14] Nemes R, Lengyel S, Nagy G, Hampton DR, Gray M, Renew JR, et al. Ipsilateral and simultaneous comparison of responses from Acceleromyography- and Electromyography-based neuromuscular monitors. Anesthesiology. 2021;135(4):597–611. 10.1097/ALN.0000000000003896. 34329371 10.1097/ALN.0000000000003896

[15] Stouffs A, Ibsen A, Jamart J, Dubois V, Dubois PE. Philips intellivue NMT module: variability of initial measurements. J Clin Monit Comput. 2018;32(5):965–6. 10.1007/s10877-017-0079-y. 29150822 10.1007/s10877-017-0079-y

[16] Stewart PA, Freelander N, Liang S, Heller G, Phillips S. Comparison of electromyography and kinemyography during recovery from non-depolarising neuromuscular Blockade. Anaesth Intensive Care. 2014;42(3):378–84. 24794479 10.1177/0310057X1404200316

[17] Bland JM, Altman DG. Measuring agreement in method comparison studies. Stat Methods Med Res. 1999;8(2):135–60. 10.1177/096228029900800204. 10501650 10.1177/096228029900800204

[18] Kirmeier E, Eriksson LI, Lewald H, Jonsson Fagerlund M, Hoeft A, Hollmann M, et al. Post-anaesthesia pulmonary complications after use of muscle relaxants (POPULAR): a multicentre, prospective observational study. Lancet Respir Med. 2019;7(2):129–40. 10.1016/S2213-2600(18)30294-7. 30224322 10.1016/S2213-2600(18)30294-7

[19] Kim NY, Koh JC, Lee KY, Kim SS, Hong JH, Nam HJ, et al. Influence of reversal of neuromuscular Blockade with Sugammadex or neostigmine on postoperative quality of recovery following a single bolus dose of Rocuronium: A prospective, randomized, double-blinded, controlled study. J Clin Anesth. 2019;57:97–102. 10.1016/j.jclinane.2019.02.014. 30939422 10.1016/j.jclinane.2019.02.014

[20] Renew JR, Hex K, Johnson P, Lovett P, Pence R. Ease of application of various neuromuscular devices for routine monitoring. Anesth Analg. 2020. 10.1213/ANE.0000000000005213. 33002932 10.1213/ANE.0000000000005213

[21] Wedemeyer Z, Michaelsen KE, Jelacic S, Silliman W, Lopez A, Togashi K, et al. Accuracy and precision of three acceleromyographs, three electromyographs, and a mechanomyograph measuring the Train-of-four ratio in the absence of neuromuscular blocking drugs. Anesthesiology. 2024;141(2):262–71. 10.1097/ALN.0000000000005051. 38728090 10.1097/ALN.0000000000005051

[22] Brull SJ, Fuchs-Buder T. Accuracy and precision of acceleromyography, electromyography, and mechanomyography: time to rethink what we know. Anesthesiology. 2024;141(2):204–7. 10.1097/ALN.0000000000005054. 38980163 10.1097/ALN.0000000000005054

[23] Blobner M, Hunter JM, Meistelman C, Hoeft A, Hollmann MW, Kirmeier E, et al. Use of a train-of-four ratio of 0.95 versus 0.9 for tracheal extubation: an exploratory analysis of POPULAR data. Br J Anaesth. 2020;124(1):63–72. 10.1016/j.bja.2019.08.023. 31607388 10.1016/j.bja.2019.08.023

[24] Claudius C, Skovgaard LT, Viby-Mogensen J. Arm-to-arm variation when evaluating neuromuscular block: an analysis of the precision and the bias and agreement between arms when using mechanomyography or acceleromyography. Br J Anaesth. 2010;105(3):310–7. 10.1093/bja/aeq162. 20595196 10.1093/bja/aeq162

[25] Kalli I. Effect of surface electrode position on the compound action potential evoked by ulnar nerve stimulation during isoflurane anaesthesia. Br J Anaesth. 1990;65(4):494–9. 10.1093/bja/65.4.494. 2104529 10.1093/bja/65.4.494

[26] Bowdle A, Bussey L, Michaelsen K, Jelacic S, Nair B, Togashi K, et al. Counting train-of-four twitch response: comparison of palpation to mechanomyography, acceleromyography, and electromyography. Br J Anaesth. 2020;124(6):712–7. 10.1016/j.bja.2020.02.022. 32228867 10.1016/j.bja.2020.02.022

[27] Epstein RH, Perez OF, Hofer IS, Renew JR, Nemes R, Brull SJ. Validation of a convolutional neural network that reliably identifies electromyographic compound motor action potentials following train-of-four stimulation: an algorithm development experimental study-Reply to: Br J Anaesth Open. 2024:100264. BJA Open. 2024;9:100265. 10.1016/j.bjao.2024.100265 10.1016/j.bjao.2024.100265 PMC10909691 38440054

