# Supplementary material for: Precision of electromyography according to the calibration approach of neuromuscular monitoring: a randomised prospective agreement study
Source: J Clin Monit Comput. 2025 May 28;39(5):1047–56. doi: 10.1007/s10877-025-01304-z (PMC12474677; doi:10.1007/s10877-025-01304-z)
Supplement: Supplementary file 1 — Supplementary Material 1 [file 10877_2025_1304_MOESM1_ESM.docx]

**Supplemental Digital Content**

**Precision of electromyography according to the calibration approach of neuromuscular monitoring: A randomised prospective agreement study**

**Flora T Scheffenbichler^1^, Bernhard Ulm^1,2^, Laura Borgstedt^2^, Anna Scholze^2^, Nadine Kretsch^2^, Magdalena Marb^1^, Nadine Zia^1^, Viola Friedrich^1^, Stefan J** Schaller**^3^, Bettina** Jungwirth**^1^, Manfred** Blobner **^1,3^**

**^1^ Department of Anaesthesiology and Intensive Care Medicine, Ulm University, Ulm, Germany
^2^ Department of Anaesthesiology and Intensive Care Medicine, TUM School of Medicine and Health, Technical University of Munich, Munich, Germany
^3^ Medical University of Vienna, Department of Anaesthesiology, Intensive Care Medicine and Pain Medicine, Division of General Anaesthesia and Intensive Care Medicine, Vienna, Austria**

**Corresponding author:**

Professor Manfred Blobner, MD
Department of Anaesthesiology and Intensive Care Medicine, Ulm University
Albert-Einstein-Allee 23, 89081 Ulm, Germany
E-Mail: [manfred.blobner@uni-ulm.de](mailto:manfred.blobner@uni-ulm.de), phone:+49-89-41407206

Table of contents

[Supplemental Methods 3](#_Toc197365838)

[Supplemental statistical analysis 3](#_Toc197365839)

[Supplemental results 3](#_Toc197365840)

[Supplemental Tables 4](#_Toc197365841)

# Supplemental Methods

## Supplemental statistical analysis

**For the secondary endpoint time difference upon reaching a TOF ratio of 0.9 or 0.95, respectively, we subtracted the time achieved using the investigational calibration from that using best practice calibration. Therefore, positive time differences indicate that the TOF ratio measured using the respective investigational calibration reached values >0.9 or 0.95 later than those of the best practice calibration.** The time difference to achieve neuromuscular recovery, recall of pain and recall of involuntary movements at anaesthesia induction were analysed using a Kruskal-Wallis test.

For an exploratory analysis, we investigated the average TOF ratio when the control or investigational calibration group, respectively, reached a TOF ratio of 0.9 for the first time. In addition, gauge standard deviation was calculated for each calibration approach.

# Supplemental results

When the TOF ratio reached 0.9 for the first time in the control group, the average TOF ratio for the investigational calibration groups was 0.89 ± 0.07 for calibration before induction, 0.91 ± 0.07 for calibration during anaesthesia induction, and 0.90 ± 0.07 for EMG without calibration.

On the other hand, when the investigational calibration approach reached 0.9 the average TOF ratio of control calibration was 0.88 ± 0.10 for calibration before induction, 0.87 ± 0.05 for calibration during induction and 0.82 ± 0.19 for uncalibrated EMG.

# Supplemental Tables

| **Supplemental Table 1.** Guidelines for Reporting Reliability and Agreement Studies (GRRAS)^1^ Checklist | | | |
| --- | --- | --- | --- |
| **Section** | **Item #** | **Checklist item** | **Reported on page #** |
| **Title/Abstract** | 1 | Identify in title or abstract that interrater/intrarater  reliability or agreement was investigated. | 1 (title) |
| **Introduction** | 2 | Name and describe the diagnostic or measurement device of interest explicitly. | 3,4 |
|  | 3 | Specify the subject population of interest. | 3 |
|  | 4 | Specify the rater population of interest (if applicable). | n/a |
|  | 5 | Describe what is already known about reliability and  agreement and provide a rationale for the study (if applicable). | 3 |
| **Methods** | 6 | Explain how the sample size was chosen. State the determined number of raters, subjects/objects, and replicate observations. | 7 |
|  | 7 | Describe the sampling method. | 5-7 |
|  | 8 | Describe the measurement/rating process (e.g. time interval between repeated measurements, availability  of clinical information, blinding). | 5-7 |
|  | 9 | State whether measurements/ratings were conducted independently. | 5 |
|  | 10 | Describe the statistical analysis. | 6,7 |
| **Results** | 11 | State the actual number of raters and subjects/objects  which were included and the number of replicate observations which were conducted. | 9 (Fig. 2) |
|  | 12 | Describe the sample characteristics of raters and  subjects (e.g. training, experience). | n/a |
|  | 13 | Report estimates of reliability and agreement including measures of statistical uncertainty. | 10-11, Fig. 3 |
| **Discussion** | 14 | Discuss the practical relevance of results. | 15-17 |
| **Auxiliary material** | 15 | Provide detailed results if possible (e.g. online). | Supplement |

**1 Kottner J, Audige L, Brorson S, et al. Guidelines for Reporting Reliability and Agreement Studies (GRRAS) were proposed. J Clin Epidemiol 2011; 64: 96-106**

| **Supplemental Table 2.** Characteristics of study cohort | | | | |
| --- | --- | --- | --- | --- |
| Characteristics | Before induction  (n=20) | During induction  (n=20) | Uncalibrated  (n=19) | All  (n=59) |
| Age  Female sex  Weight (kg)  ASA physical status  I  II  III  Rocuronium (mg)  Sugammadex (mg) | 43 (34-62)  11  69 (63-80)  10  8  2  43 (40-45)  142 (131-160) | 48 (28-63)  7  79 (60-86)  10  7  3  46 (42-49)  158 (120-174) | 38 (26-64)  7  73 (68-80)  9  8  2  44 (39-46)  146 (138-161) | 44 (29-63)  25  74 (63-82)  29  23  7  45 (40-48)  150 (131-165) |
| Surgical service  General  Urology  Orthopedics | 3  4  13 | 2  8  10 | 4  2  13 | 9  14  36 |
| Values indicate numbers unless otherwise specified. Data are presented as median (interquartile range) or number. ASA = American society of Anesthesiologists. Continuous data are presented as median (interquartile range). | | | | |

| **Supplemental Table 3.** Gauge standard deviation of baseline train-of-four ratios prior to rocuronium administration | | | | |
| --- | --- | --- | --- | --- |
|  | Before induction  (n=20) | During induction  (n=20) | Uncalibrated  (n=19) | Control  (n=59) |
| Gauge standard deviation | 0.04 ± 0.010 | 0.06 ± 0.006 | 0.07 ± 0.010 | 0.03 ± 0.003 |

| **Supplemental Table 4.** Bias, lower and upper limits of agreement together with the 95% confidence interval as evaluated in Bland Altman analysis. | | | | |
| --- | --- | --- | --- | --- |
|  | TOF-R | Bias | Lower LoA | Upper LoA |
| Before induction | Baseline  20-39  40-59  60-79  ≥80  All (≥20) | 0.006 [-0.002 to 0.014]  0.055 [0.041 to 0.069]  0.041 [0.024 to 0.057]  0.019 [0.007 to 0.031]  -0.002 [-0.005 to 0.002]  0.014 [0.010 to 0.018] | -0.080 [-0.094 to -0.067]  -0.189 [-0.213 to -0.165]  -0.249 [-0.277 to -0.221]  -0.227 [-0.248 to -0.207]  -0.133 [-0.139 to -0.127]  -0.186 [-0.193 to -0.180] | 0.093 [0.079 to 0.106]  0.300 [0.276 to 0.323]  0.330 [0.302 to 0.359]  0.265 [0.245 to 0.286]  0.129 [0.123 to 0.135]  0.215 [0.208 to 0.222] |
| During induction | Baseline  20-39  40-59  60-79  ≥80  All (≥20) | -0.000 [-0.009 to 0.009]  0.012 [0.000 to 0.023]  0.012 [-0.001 to 0.025]  0.003 [-0.008 to 0.014]  0.000 [-0.003 to 0.003]  0.003 [0.000 to 0.006] | -0.099 [-0.114 to -0.084]  -0.166 [-0.186 to -0.147]  -0.186 [-0.208 to -0.164]  -0.188 [-0.207 to -0.169]  -0.112 [-0.116 to -0.107]  -0.140 [-0.145 to -0.135] | 0.098 [0.083 to 0.113]  0.190 [0.170 to 0.209]  0.210 [0.188 to 0.232]  0.194 [0.175 to 0.212]  0.112 [0.107 to 0.117]  0.146 [0.141 to 0.152] |
| Uncalibrated | Baseline  20-39  40-59  60-79  ≥80  All (≥20) | 0.002 [-0.006 to 0.011]  0.029 [0.017 to 0.040]  0.054 [0.038 to 0.069]  0.042 [0.028 to 0.056]  0.008 [0.004 to 0.012]  0.020 [0.016 to 0.024] | -0.082 [-0.097 to -0.067]  -0.169 [-0.189 to -0.150]  -0.174 [-0.201 to -0.147]  -0.192 [-0.216 to -0.168]  -0.135 [-0.142 to -0.128]  -0.159 [-0.165 to -0.152] | 0.086 [0.072 to 0.101]  0.227 [0.207 to 0.247]  0.282 [0.255 to 0.309]  0.276 [0.252 to 0.300]  0.151 [0.144 to 0.157]  0.198 [0.192 to 0.205] |
| LoA, limits of agreement; TOF-R, train-of-four-ratio. | | | | |

| **Supplemental Table 5.** Median and interquartile ranges of time to reach a TOF ratio of 0.9 and 0.95, respectively. | | | | |
| --- | --- | --- | --- | --- |
| Characteristics | Before anaesthesia induction  (n=20) | During anaesthesia induction  (n=20) | Uncalibrated  (n=19) | p-value |
| Intervention reaching TOF ratio >0.9 prior to control^1^  Time difference (s) to reach a TOF ratio ≥0.9  Intervention reaching TOF ratio ≥0.95 prior to control^1^  Time difference (s) to reach a TOF ratio ≥0.95 | 8  -20 (-180-420)  7  -20 (-250-150) | 13  60 (-85-205)  13  60 (-70-325) | 11  40 (-50-285)  12  40 (-125-320) | 0.4^1^  0.8^2^  0.2^1^  0.7^2^ |
| The time difference was calculated subtracting the time to achieve the threshold in question in the control EMG from the intervention EMG (control– intervention time). 20 seconds was the minimum difference corresponding to one TOF measurement. Comparisons were made using Kruskal Wallis rank sum test.  ^1^Pearson’s Chi-squared test; ^2^Kruskal-Wallis rank sum test. | | | | |
